# Improving physician hand hygiene compliance using behavioural theories: a study protocol

**DOI:** 10.1186/1748-5908-8-16

**Published:** 2013-02-04

**Authors:** Janet E Squires, Kathryn N Suh, Stefanie Linklater, Natalie Bruce, Kathleen Gartke, Ian D Graham, Alan Karovitch, Joanne Read, Virginia Roth, Karen Stockton, Emma Tibbo, Kent Woodhall, Jim Worthington, Jeremy M Grimshaw

**Affiliations:** 1 Clinical Epidemiology Program, Ottawa Hospital Research Institute, Ottawa, ON, Canada; 2 School of Nursing, Faculty of Health Sciences, University of Ottawa, Ottawa, ON, Canada; 3 Department of Medicine, The Ottawa Hospital/University of Ottawa, Ottawa, ON, Canada; 4 Infection Prevention and Control, The Ottawa Hospital, Ottawa, ON, Canada; 5 Department of Surgery, The Ottawa Hospital/University of Ottawa, Ottawa, ON, Canada; 6 Ambulatory Care/Logistical Services, The Ottawa Hospital, Ottawa, ON, Canada; 7 Quality and Patient Safety, The Ottawa Hospital, Ottawa, ON, Canada; 8 Perioperative and Regional Cancer Programs, The Ottawa Hospital, Ottawa, ON, Canada; 9 Medical Affairs, Quality and Patient Safety, The Ottawa Hospital, Ottawa, ON, Canada

## Abstract

**Background:**

Healthcare-associated infections affect 10% of patients in Canadian acute-care hospitals and are significant and preventable causes of morbidity and mortality among hospitalized patients. Hand hygiene is among the simplest and most effective preventive measures to reduce these infections. However, compliance with hand hygiene among healthcare workers, specifically among physicians, is consistently suboptimal. We aim to first identify the barriers and enablers to physician hand hygiene compliance, and then to develop and pilot a theory-based knowledge translation intervention to increase physicians’ compliance with best hand hygiene practice.

**Design:**

The study consists of three phases. In Phase 1, we will identify barriers and enablers to hand hygiene compliance by physicians. This will include: key informant interviews with physicians and residents using a structured interview guide, informed by the Theoretical Domains Framework; nonparticipant observation of physician/resident hand hygiene audit sessions; and focus groups with hand hygiene experts. In Phase 2, we will conduct intervention mapping to develop a theory-based knowledge translation intervention to improve physician hand hygiene compliance. Finally, in Phase 3, we will pilot the knowledge translation intervention in four patient care units.

**Discussion:**

In this study, we will use a behavioural theory approach to obtain a better understanding of the barriers and enablers to physician hand hygiene compliance. This will provide a comprehensive framework on which to develop knowledge translation interventions that may be more successful in improving hand hygiene practice. Upon completion of this study, we will refine the piloted knowledge translation intervention so it can be tested in a multi-site cluster randomized controlled trial.

## Background

Healthcare-associated infections are ranked by the World Health Organization as one of the top 10 causes of hospital deaths worldwide [1]. In Canada, they are the most common serious complication of hospitalization, affecting 10% of all patients in acute-care hospitals [2] and are the fourth leading cause of death [3]. Healthcare-associated pathogens that can lead to infection are transmitted through direct and indirect contact, droplets, air, and the contaminated hands of healthcare workers (HCWs), the latter being the most common vehicles of transmission in most settings [4]. Microorganisms are known to survive on hands for up to 60 minutes following contact with a patient or contaminated surface. Hand contamination increases with increasing time spend providing direct patient care, in the absence of appropriate hand hygiene [4].

Hand hygiene, defined as the act of washing one’s hands with soap and water or disinfecting them with an antiseptic agent, is the single most successful and cost-effective means of preventing healthcare-associated infections, as well as an effective means of preventing illness in the community that may lead to hospitalization [5-8]. Hand hygiene, before and after all patient or patient environment contact, before aseptic procedure, and/or after bodily fluid exposure, is recommended in all published infection control and public health guidelines and is considered the standard of care for all HCWs [5-10]. Yet many studies document that HCWs’ compliance with hand hygiene recommendations is consistently less than 50% [6,11-15], with compliance among physicians routinely lower than that of other HCWs [16-19]. Locally, at the Ottawa Hospital (proposed for this study), physician hand hygiene compliance rates have increased significantly from 14–17% between 2004–6 to 65–69% in March 2011. In spite of this increase, however, physician compliance continues to lag behind that of most other occupational groups and is below the corporate goal of 80%.

### Interventions to improve hand hygiene compliance

In 2010, Gould and colleagues [20] conducted an update of the 2007 Cochrane systematic review [21] on interventions to improve hand hygiene compliance in patient care. A total of four studies are included in the review: two from the original review and two from the update. Interventions tested included substitutions of products and different multifaceted campaigns that involved different levels of involvement by HCWs. Success in improving hand hygiene compliance was inconsistent across the four studies. The authors concluded that we lack sufficient research evidence to know which strategies improve hand hygiene compliance and that robust research is needed to further explore ‘the effectiveness of soundly designed and implemented interventions to increase hand hygiene compliance’ [20]. Most recently, Fuller and colleagues conducted a cluster randomised controlled trial of a behaviourally designed feedback intervention in 60 hospital wards across England and Wales that were implementing a national hand-hygiene campaign [22]. Findings revealed that the intervention, which coupled feedback to personalized action planning (compared to routine care), produced moderate and significant sustained improvements in hand-hygiene compliance [22]. While this study indicates promising effects for the use of behaviourally designed feedback interventions to improve hand hygiene compliance, further implementation studies are required to determine the intervention’s effect in different settings and contexts.

### Barriers and enablers to physician hand hygiene compliance

Reasons for low hand hygiene compliance by HCWs, and physicians specifically, are poorly understood. Studies investigating HCWs generally have reported a range of barriers, including environmental barriers (*e.g*., lack of access to sinks, difficulty of locating products, empty dispensers, dispensers and time constraints) and personal barriers (*e.g*., attitudinal beliefs, skin irritation from repeated hand washing) [23,24]. Using a behavioural theory approach, Boscart and colleagues explored nurses’ perceived barriers and enablers to hand hygiene practice [25]. Nurses focused on immediate consequences; for example, they identified their personal safety and their families’ safety as a core source of motivation to perform hand hygiene. They also described the importance of individual feedback and self-monitoring in order to increase their performance. With respect to barriers specific to physicians, research is limited. In a recent survey of attitudes towards hand hygiene, physicians reported ‘remembering to perform hand hygiene’ and ‘high workload or feeling too rushed’ as their top barriers to hand hygiene compliance [26]. A second study, which surveyed a variety of HCWs including physicians, found environmental barriers to hand hygiene compliance to be dominant, including lack of soap, broken soap dispensers, and lack of paper towels [27]. Educational gaps in infection control training among physicians also exist [17,18,28,29]; however, strategies effective for improving infection control practices of other HCWs have had significantly less impact on physicians [30,31]. Additional barriers that are specific to physicians that have been identified/postulated include: a perception among physicians that their compliance is much better than it actually is [32,33]; the development of a more cavalier attitude towards infection control as clinical experience increases, with an associated drop in compliance rates [30,34,35]; the lack of positive role models among physicians who are part of a healthcare team [35-37]; and, the local (*e.g*., unit, hospital) culture of patient safety [38].

In summary, the barriers and enablers to physician hand hygiene compliance and effective interventions to improve their compliance have not been well explored. To our knowledge, no studies have specifically addressed physician hand hygiene compliance using a behavioural theory approach that encompasses both barrier and enabler assessment, followed by intervention design based on these assessments. Therefore, the aims of this study are, first, to identify the barriers and enablers to physician hand hygiene compliance, and then to develop and pilot a theory-based knowledge translation intervention to increase physicians’ compliance with best hand hygiene practice.

### Guiding framework

Our goal is to develop and evaluate a theory-based, knowledge translation intervention to provide practical guidance about how to improve physician hand hygiene compliance. However, the development and evaluation of complex interventions such as knowledge translation interventions raise specific methodological and conceptual challenges. We have therefore adopted the UK Medical Research Council Complex Interventions Framework (MRC Framework) [39,40], which provides an iterative phased approach to the development and evaluation of complex interventions. The MRC framework suggests that the evaluation of complex interventions should follow a sequential approach, involving:

1. Phase 0: problem and contextual assessment, and development of the theoretical basis for an intervention;

2. Phase 1: definition of components of the intervention (using modeling or simulated techniques and qualitative methods);

3. Phase 2: exploratory studies to further develop the intervention and plan a definitive evaluative study (using a variety of methods);

4. Phase 3: definitive evaluative studies (using quantitative evaluative methods, predominantly randomized designs); and,

5. Phase 4: studies evaluating the sustainability of complex interventions.

Campbell and colleagues suggested that Phases 0–2 should be considered part of a larger iterative activity rather than as sequential studies, and highlighted that the insights gained during these early phases can make a valuable contribution to the development of the basic science of knowledge translation [40]. Based upon current systematic reviews, it appears that many knowledge translation studies have involved definitive trials, with little evidence of preceding theoretical or modeling research [41]. As a result, the interpretation of the current evidence base on the effectiveness and efficiency of different strategies is problematic because we lack a theoretical base for conceptualizing decision-making and behaviour change processes in different stakeholder groups. As a result, it is difficult to apply this evidence across a variety of health settings because we cannot identify which interventions are most likely, in particular settings, to be effective or efficient in improving quality. Further, we have little understanding of the causal mechanisms of different interventions. In this study, we will adopt an iterative approach (as suggested by Campbell and colleagues [40]) to the development and evaluation of a knowledge translation intervention to improve physician hand hygiene compliance.

## Methods

In this study, we define hand hygiene as: any action of hand cleaning that removes visible soil and removes and kills any microorganisms on the hands. We propose to use state of the art approaches from knowledge translation science to, first, identify key factors that act as barriers and enablers to best hand hygiene practice by physicians and then develop and pilot a knowledge translation intervention focused on improving hand hygiene compliance by physicians that is based on the identified barriers and enablers.

The study will be subdivided into three phases to be executed over one year:

Phase 1: identification of barriers and enablers to hand hygiene compliance by physicians. This will include: key informant interviews with physicians and residents; nonparticipant observation of physician/resident hand hygiene behaviours during regular hospital hand hygiene audit sessions; and focus groups with hand hygiene experts (this Phase is equivalent to Phase 0 in MRC framework).

Phase 2: intervention mapping to develop a theory-based knowledge translation intervention to improve physician hand hygiene compliance (this Phase is equivalent to Phase 1 in MRC framework).

Phase 3: implementation and pilot of the knowledge translation intervention (this Phase is equivalent to Phase 2 in MRC framework).

### Phase 1: Identification of barriers and enablers to physician hand hygiene compliance

The purpose of Phase 1 is to identify and describe physicians’ beliefs and attitudes about hand hygiene and their perceptions of the multi-level factors that influence this behaviour. This will allow us to identify determinants of the evidence-practice gap (*i.e.*, why physician hand hygiene compliance is inconsistent); specific behaviors that need changing in order to increase physician hand hygiene compliance; and the specific targets to be addressed by our theory-based knowledge translation intervention. This necessary preliminary work will generate a thorough understanding of physicians’ perceptions of the multi-level determinants of the behaviours required to improve physician hand hygiene compliance. Our data will come primarily from semi-structured interviews with physicians and residents. We will supplement this data with non-participant observation of physicians’ and residents’ hand hygiene practices (during regularly conducted hospital hand hygiene audit sessions) and focus groups with hand hygiene experts.

### Semi-structured interviews with physician and residents

#### Study population and sampling

Key informants will comprise staff physicians and residents in medicine and surgery at two geographical campuses from a large Canadian urban hospital. Lists of all eligible physicians and residents (divided by campus [n = 2] and specialty [n = 2, medicine and surgery]) will be obtained. From this master list, six new lists will be created for sampling purposes: (1) physicians medicine campus 1, (2) physicians medicine campus 2, (3) physicians surgery campus 1, (4) physicians surgery campus 2, (5) residents medicine, and (6) residents surgery. Residents will not be separated by campus as they work equally across campuses. A quasi-experimental sampling strategy will be used to select key informants from each list. The lists of potential key informants will be arranged in ascending order with respect to the informant’s name (first, last name), with each informant being assigned a number. The first key informant will be chosen at random, and the subsequent key informants will then be selected according to regular intervals, known as periods. A period is determined by dividing the population size (N on the list) by the desired sample size (n that is 5 or 10, depending on the group). The Associate Director of Infection Prevention and Control of the hospital will inform the chosen key informants via email that they have been selected for the study. Key informants who agree to participate will be instructed to contact the study research assistant (SL), at which time they will be provided additional details on the study. If they remain willing to participate after this initial contact, a time will be arranged to conduct the interview. This selection process will be repeated until the required sample size is achieved.

In qualitative research, there are no hard and fast rules about sample sizes. Determining adequate sample size is ultimately a matter of judgment and experience in evaluating the quality of the information collected against the uses to which it will be put, the particular research method and purposeful sampling strategy employed, and the research products intended [42]*.* We will use the concept of ‘data saturation’ to determine when we have interviewed a sufficient number of key informants. In other words, we will conduct interviews until no new barriers or enablers to physician hand hygiene compliance are being identified. Based on the diversity of our sample, *i.e*., physicians with an increasing degree of clinical experience (residents and staff physicians) and two medical specialties requiring different clinical practices (medicine and surgery), we propose to conduct a minimum of 40 interviews (divided equally between specialty and campus, with equal representation among residents and staff physicians; see Table 1). We will employ a stopping criterion of three interviews. The stopping criterion refers to the number of interviews to be conducted per sampled group, without new themes or concepts emerging, before we can conclude that data saturation has been reached [43].

**Table 1:** Key informant interview sample

**Key informant group**	**Minimum number of interviews to be conducted**				
	**Medicine**		**Surgery**		**Total Interviews**
	**Campus 1**	**Campus 2**	**Campus 1**	**Campus 2**	
Residents	10		10		20
Staff Physicians	5	5	5	5	20
Total Interviews					40

#### Data collection

We will use qualitative methods to identify barriers and enablers to physician hand hygiene compliance or non-compliance that can be addressed in a knowledge translation intervention to change (improve) this behaviour. We will conduct semi-structured interviews with key informants – *i.e*., physicians and residents. A semi-structured interview guide, informed by the Theoretical Domains Framework (TDF) [44,45], has been developed and pilot-tested for this purpose (See Additional File 1). The TDF is a behavior change framework that was developed using a systematic consensus approach to simplify psychological theories relevant to behavior change. The framework includes 14 ‘theoretical domains’ derived from 128 constructs from 33 health and social psychology theories that may explain health-related behavior change. The 14 domains offer wide coverage of the potential multi-level determinants of health-related behavior and guide the use of broad prompts that enable interviewees to consider a wide range of possibilities without asking leading questions. Identification of the 14 domains and definitions of each domain is summarized in Table 2. The TDF can be used to inform the choice of potential behavior change techniques to develop interventions as well as to investigate determinants of behavior [44-46]. The TDF-informed interview guide will be used in this project to probe the key informants about reasons they do or don’t adhere to best hand hygiene practices consistently in their clinical practice. This will allow us to identify key beliefs from different domains that could be targeted by knowledge translation interventions to improve their compliance.

**Table 2:** Domains in the theoretical domains framework

**Theoretical Domain**	**Definition [**[44]**]**
Knowledge	An awareness of the existence of something
Skills	An ability or proficiency acquired through practice
Social/Professional Role and Identity	A coherent set of behaviours and displayed personal qualities of an individual in a social or work setting
Beliefs about capabilities	Acceptance of the truth, reality or validity about an ability, talent or facility that a person can put to constructive use
Optimism	The confidence that things will happen for the best or that desired goals will be attained
Beliefs about consequences	Acceptance of the truth, reality or validity about outcomes of a behaviour in a given situation
Reinforcement	Increasing the probability of a response by arranging a dependent relationship, or contingency, between the response and a given stimulus
Intentions	A conscious decision to perform a behaviour or a resolve to act in a certain way
Goals	Mental representations of outcomes or end states that an individual wants to achieve
Memory, attention and decision processes	The ability to retain information, focus selectively on aspects of the environment and choose between two or more alternatives
Environmental context and resources	Any circumstance of a person’s situation or environment that discourages or encourages the development of skills and abilities, independence, social competence, and adaptive behaviour
Social influences	Those interpersonal processes that can cause individuals to change their thoughts, feelings or behaviours
Emotion	A complex reaction pattern, involving experiential, behavioural and physiological elements, by which the individual attempts to deal with a personally significant matter or event
Behavioural regulation	Anything aimed at managing or changing objectively observed or measured actions

We chose to use semi-structured key informant interviews using a theory-based interview guide for three reasons. First, this approach allows participants to respond freely, to illustrate concepts and to present individual perspectives that the interviewer can probe further [47]. Second, a semi-structured interview guide will increase the likelihood that busy participants cover the topics of interest in an efficient manner. Third, such a guide facilitates flexibility, such that an interviewer may explore in greater depth the issues that may arise in the interview that are not addressed by the interview guide [48,49]*.*

The study research assistant (SL), who has been trained in interviewing skills using mock interviews to ensure she is comfortable with the interview guide, will conduct the interviews. Interviews will be audio-recorded (with the participant’s consent) and are expected to last approximately 15–20 minutes. Before beginning each interview, informed consent will be obtained from each key informant. Interviews will be conducted by telephone or in-person, according to the key informant’s preference. Key informants will be given a $20 coffee-shop gift card in appreciation of their time.

#### Data analysis

The interviews will yield a large quantity of data. To monitor the progress of the interviews and permit follow-up of issues that may emerge from the data, interviewing, transcription and analysis will occur concurrently in this phase of the study. The recordings will be transcribed verbatim and verified by the interviewer prior to analysis. Data will be analyzed using NVivo 9, a standard qualitative software program [50]. Interview transcript analysis will involve the following three steps [51,52]:

#### Coding interview transcripts

To facilitate analysis and ensure consistency in coding, two coders will independently code responses from the first two interviews into the TDF theoretical domains (Table 2) and then compare their results to develop a coding scheme. Thereafter, all coding will be guided by the coding scheme. The use of a coding scheme in qualitative analysis is important to reducing subjective bias [53]. The same two coders will code all remaining interviews. The coders will meet frequently (after every five interviews) to review their coding and seek consensus. Coder reliability between the two coders will be assessed using a method proposed by Miles and Huberman [54]: Coder reliability = number of agreements/(total number of agreements + disagreements). In instances of disagreement, we will discuss the rationale behind the coding to come to consensus on an agreed code. Level of agreement between the two coders should meet or exceed 70%, the level of agreement that is considered acceptable to ensure confidence in the coding process [54]. If agreement is not achieved, the text will be allocated into all domains identified by both coders.

#### Generating specific beliefs

Statements describing specific underlying beliefs will be generated for each response within each theoretical domain (of the TDF), by one team member (SL), which will be double-checked by the second coder and also a researcher (JS, JG). We will generate specific beliefs by professional group and specialty: physicians-medicine; physicians-surgery; residents-medicine; and residents-surgery. A specific belief refers to a collection of responses with a similar underlying theme that suggests a problem and/or influence of the belief on the target behaviour [51,52,55]. A frequency count (*i.e*., number of key informants)/belief statement will be derived across the interviews in the four subgroups identified above.

#### Identifying relevant theoretical domains

In line with guidelines previously published [51,52,55], we will judge which of the 14 TDF theoretical domains are relevant to our target behavior. Domains that contain specific beliefs (identified in step two above) that might act as potential barriers or enablers to changing physician hand hygiene behaviour and fulfill the following three criteria will be judged as relevant: relatively high frequency of specific beliefs; presence of conflicting beliefs; and evidence of strong beliefs that may impact on the behavior (our hand hygiene specialist [KS] on the team will help interpret the importance of specific beliefs from a clinical perspective). All three criteria will be considered simultaneously in determining relevance of the theoretical domains.

Findings from this analysis will be used in two ways. First, we will use the data to develop a rich description of physicians’ views on the factors influencing their hand hygiene compliance. Second, we will use the findings to develop a theory-based knowledge translation intervention to improve the behaviour (see Phase 2).

#### Non-participant observation

Concurrent with the key informant interviews, we will conduct non-participant observation to identify any additional perceived (by the observer) barriers and enablers to physician hand hygiene compliance not identified in the semi-structured interviews conducted with physician and residents. We will collect this data by observing physicians during routine hospital hand hygiene audit sessions, which includes interaction between the auditor and physicians/residents. We will observe both the environmental context of the interactions as well as the process of the interactions. The assessment will be guided by an observation checklist (Table 3). Two team members (SL, JS) will conduct the non-participant observation by accompanying a hospital-trained auditor on a minimum of four routine sessions; a minimum of two sessions at each of the two hospital campuses sampled in the study will be attended. Field notes will be compiled, transcribed and then analyzed using qualitative content analysis. Qualitative content analysis focuses on unique themes found within the text, rather than the statistical significance of key words or concepts as in quantitative content analysis [56]. We will follow the same principle of ‘data saturation’ used with the interviews, observing audit sessions until no new barriers or enablers to physician compliance with hand hygiene are identified [43,57]. Prior to conducting the non-participant observation, the two data collectors will attend an auditor training session to gain further insight into the process.

**Table 3:** Non-participant observation checklist

**Observation of the environmental context of interactions**	**Observation of the process of the interaction**
Who is present during the interaction?	Who speaks to whom and about what? (*i.e*., the auditor and physician interaction)
Physical space (use a diagram to depict the set up of space and how people, patients and sinks/alcohol hand rub dispensers are positioned in that space)	How are concerns responded to?
Describe how patient care is delivered in the setting (*i.e*., is this a shared room? Are other patients receiving care at the same time?)	Are the physicians receiving feedback from the auditors?
Location of the auditor in relation to the physician being observed	If feedback is given, how do the physicians respond?
Describe the length of the interaction	Whenever possible, capture verbatim accounts between individuals present

#### Focus groups with hand hygiene experts

We will also conduct focused discussions with hand hygiene experts (*e.g*., hand hygiene auditors, infection control specialists, and members of senior management) using a modified version of the TDF-based interview guide used with the key informants. The Associate Director of Infection Prevention and Control (KS) will request volunteers for the focus groups from each campus. We will target 6–8 participants per focus group and a total of two focus groups (60–90 minutes each; one focus group per campus). Two members of the research team (SL, JS) will conduct the focus groups with one team member acting as a moderator and the other as an observer/recorder. Each focus group will be audio-recorded and transcribed. The transcripts will be checked for accuracy before analysis. Analysis will follow the same procedure outlined previously for key informant interviews (double coding of interview transcripts, generation of specific beliefs, and identification of relevant theoretical domains).

#### Phase 2: Design of a theory-based knowledge translation intervention to improve physician hand hygiene compliance

Using the data obtained in Phase 1 (identification of barriers and enablers to physician hand hygiene compliance), we will develop a theory-based knowledge translation intervention that encompasses behavior change techniques to overcome the identified barriers and enhance the enablers to hand hygiene compliance by physicians. The intervention will be developed using intervention mapping, a formal systematic process for building interventions based upon identified barriers and enablers to behaviour change [58]*.* In this process, the TDF theoretical domains identified as relevant in Phase 1 (in the interviews with physicians and focus groups with hand hygiene experts) will be prioritized. We will also consider any additional barriers identified in the non-participant observation sessions. A range of behaviour change techniques will be selected to address the most important domains (barriers to hand hygiene compliance). We will use the Behavior Change Matrix [59], developed by Michie and colleagues, in this process. The Behavior Change Matrix consists of a list of 53 effective behaviour change techniques, determined based on expert consensus and systematic review, mapped onto the TDF domains. This approach simplifies the diverse science of behaviour change into a taxonomy of behaviour change techniques that can be used to design knowledge translation interventions with well-articulated causal pathways [59].

The behavior mapping process explicates the program logic of the resulting intervention by explicitly linking behaviour change techniques to the barriers that participants target. The intervention techniques we choose and their delivery in the pilot (Phase 3) will be based upon: empirical evidence and expert consensus of the effectiveness of the behavior change techniques; what is likely to be feasible in our specific context; and what is locally relevant and acceptable. The investigative team, which includes researchers and clinicians, will be involved in the design of the intervention. We will hold a series of team meetings to carry out the mapping process and to design and plan the implementation of the intervention.

#### Phase 3: Implementation and pilot of the intervention

We will pilot the theory-based knowledge translation intervention determined in Phase 2 of our work in four patient care units at the same large urban hospital used to collect our data on barriers and enablers. Routine hand hygiene audits collected by the hospital will be used to determine which units are ‘low performing.’ We will randomly select for the pilot, from all ‘low performing’ medical and surgical units, two medical and two surgical units (divided equally between campuses). We will assess effectiveness of the intervention using pre- and post-hand hygiene audits (counts of physicians/residents who washed their hands in accordance with current guidelines), which will be collected by hospital-trained auditors. We will report pre- and post-audit numbers and perform a chi-square test to examine differences between proportions of physicians/residents performing appropriate hand hygiene pre- and post- intervention if there is sufficient sample size (*i.e*., sufficient numbers of physicians/residents audited on each unit). An important element of our evaluation in Phase 3 will be a process evaluation to understand what aspects of the intervention did and did not work. The process evaluation will involve interviews with clinicians (*e.g*., physicians, residents, nurses) on the pilot units as well with hand hygiene experts across the hospital. We will conduct interviews with up to five residents and staff physicians per piloted unit. In the focus group, we will target six to eight hand hygiene experts [60]. We will follow the same process for conducting the focus groups as previously outlined. All process interviews and focus groups will be analyzed using thematic content analysis.

#### Ethics

Ethical approval for this study was obtained from the Ottawa Hospital Research Ethics Board (protocol # 2012040801H). Operational approval was obtained from The Ottawa Hospital.

## Discussion

Addressing physician behaviour is especially important given the role model and leadership roles that physicians play in healthcare settings. A better understanding of the rationale for specific physician behaviours related to hand hygiene will provide a more comprehensive framework on which to develop interventions that have a better than random chance of being successful in effecting change in this group. This can be achieved through the application of a behavioural theory approach such as that which will be applied in this study. The study presented in this protocol is based on the TDF that is derived from theories of behaviour [44]. Conducting semi-structured interviews (based on the TDF) to identify important theoretical domains to behaviour change is an approach that has rarely been used before, and to our knowledge has never been used with respect to physicians’ use of hand hygiene practice.

This study corresponds to specific aspects of the UK’s MRC framework for developing complex interventions [39,40], namely, phases 0 (problem and contextual assessment and development of the theoretical basis for an intervention), 1 (definition of components of the intervention), and 2 (exploratory studies to develop further the intervention and plan a definitive evaluative study). It is now argued that these phases should be considered as part of a larger iterative activity (as will be done in this study), rather than as sequential studies [40]. It is also believed that insights gained during these early phases of intervention development and implementation can make valuable contributions to the science of knowledge translation [40]. Upon completion of this study, we will know the barriers and enablers to physician hand hygiene compliance and have developed and piloted a theory-based knowledge translation intervention that addresses these barriers/enablers. In the future, we plan to refine the piloted intervention so it can be tested in a multi-site cluster randomized controlled trial.

A limitation to this study will be measurement of the target behaviour (*i.e*., measurement of hand hygiene compliance). There are three main methods for measuring hand hygiene performance, each of which has advantages and disadvantages: directly observing, measuring product use, and self-report. Direct observation of hand hygiene behavior of HCWs chosen for this study is considered the ‘gold standard’ of measurement methods. It allows you to see which hand hygiene products are used, the thoroughness of cleansing, and importantly, whether the staff is performing hand hygiene whenever there is an opportunity to do so. This method also allows observers to give prompt feedback about whether improvement is needed. On the other hand, direct observation is also labor intensive and expensive, requiring the careful selection and training of those who will observe and record data. It can also influence the behavior of those who know they are being observed, which is a potential bias in our study. The success of this method depends on the accurate calculation of adherence rates, the careful training of data collectors, and the data collectors’ uses of clear, easy-to-understand forms. Therefore, in an attempt to limit bias, we will use hospital-trained auditors.

## Competing interests

The authors declare that they have no competing interests.

## Authors’ contributions

JES, KS, and JMG conceived the study and secured its funding. JES and SL drafted the manuscript. All authors provided input into the protocol, critical feedback on the manuscript, and approved the final manuscript.

## Supplementary Material

Additional file 1 Interview Guide. Click here for file

## References

[B1] World Health Organization Patient Safety WHO Guidelines on Hand Hygiene in Health Care: First Global Patient Safety Challenge, Clean Care is Safer Care 2009 Geneva Switzerland: World Health Organization 23805438

[B2] Gravel D Taylor G Ofner M Johnston L Loeb M Roth VR Stegenga J Bryce E Point prevalence survey for healthcare-associated infections within Canadian adult acute-care hospitals J Hosp Infect 2007 66 243 248 10.1016/j.jhin.2007.04.008 17574304

[B3] Baker GR Norton PG Flintoft V Blais R Brown A Cox J Etchells E Ghali WA Hebert P Majumdar SR O’Beirne M Palacios-Derflingher L Reid RJ Sheps S Tamblyn R The Canadian Adverse Events Study: The incidence of adverse events among hospital patients in Canada CMAJ 2004 170 1678 1686 15159366 10.1503/cmaj.1040498 PMC408508

[B4] World Health Organization Patient Safety WHO Guidelines on Hand Hygiene in Health Care: a Summary 2009 Geneva, Switzerland: World Health Organization

[B5] Health Canada Infection Control Guidelines Handwashing, Cleaning, Disinfection and Sterilization in Health Care Can Comm Dis Rep 1998 24S8 1 66

[B6] Boyce JM Pittett D Guideline for hand hygiene in health-care settings: recommendations of the healthcare infection control practices advisory committee and the HICPAC/SHEA/APIC/IDSA hand hygiene task force Infection Control and Hospital Epidemiology 2002 23 1 45 10.1086/503164 12515399

[B7] Garner JS Favero MS Guideline for handwashing and hospital environmental control, 1985 Atlanta Centers for Disease Control and Prevention 1985 http://wonder.cdc.gov/wonder/prevguid/p0000412/p0000412.asp. 10.1017/s0195941700084022 3009347

[B8] Larson E Association for Professionals in Infection Control and Epidemiology 1992–1993, 1994 APIC Guidelines Committee APIC guideline for handwashing and hand antisepsis in health care settings Am J Infect Control 1995 23 251 269 10.1016/0196-6553(95)90070-5 7503437

[B9] Public Health Ontario Just Clean Your Hands - Your 4 Moments for Hand Hygiene 2011 Public Health Ontario: Ontario Agency for Health Protection and Promotion 7-8-2012

[B10] Centre for Disease Control Guideline for Hand Hygiene in Health-Care Settings-Recommendations of the Healthcare Infection Control Practices Advisory Committee and the HICPAC/SHEA/APIC/IDSA Hand Hygiene Task Force MMWR Morb Mortal Wkly Rep 2002 51 http://www.cdc.gov/mmwr/pdf/rr/rr5116.pdf.

[B11] Bischoff WE Reynolds TM Sessler CN Edmond MB Wenzel RP Handwashing complicance by health care workers. The impact of introducing an accessible, alcohol-based hand antiseptic Arch Intern Med 2000 160 1017 1021 10.1001/archinte.160.7.1017 10761968

[B12] Earl ML Jackson MM Rickman LS Improved rates of compliance with hand antisepsis guidelines: a three phase observational study Am J Nurs 2001 101 26 33 11279993 10.1097/00000446-200103000-00038

[B13] Moongtui W Gauthier D Turner J Using peer feedback to improve handwashing and glove usage among Thai health care workers Am J Infect Control 2000 28 365 369 10.1067/mic.2000.107885 11029136

[B14] Muto CA Sistrom MG Farr BM Hand hygiene rates unaffected by installation of dispensers of a rapidly acting hand antiseptic Am J Infect Control 2000 28 273 276 10.1067/mic.2000.103242 10840351

[B15] Rosenthal VD McCormick RD Guzman S Villamayor C Orellano PW Effect of education and performance feedback on handwashing: the benefit of administrative support in Argentinian hospitals Am J Infect Control 2003 31 85 92 10.1067/mic.2003.63 12665741

[B16] Kaplan LM McGuckin M Increasing handwashing compliance with more accessible sinks Infect Control 1986 7 408 410 3638277 10.1017/s019594170006464x

[B17] Larson EL McGinley KJ Foglia A Leyden JJ Boland N Larson J Altobelli LC Salazar-Lindo E Handwashing practices and resistance and density of bacterial hand flora on two pediatric units in Lima, Peru Am J Infect Control 1992 20 65 72 10.1016/S0196-6553(05)80003-3 1590601

[B18] Pittet D Mourouga P Perneger TV Compliance with handwashing in a teaching hospital. Infection Control Program Ann Intern Med 1999 130 126 130 10068358 10.7326/0003-4819-130-2-199901190-00006

[B19] Alberta Health Services Alberta Health ServicesHand Hygiene Compliance Provincial, Zone and Hospital/Site Report 2012 12-9-2011. 7-11-2012 http://www.albertahealthservices.ca/Publications/ahs-pub-pr-det-hh-ipc-site-q2w92q9t.pdf.

[B20] Gould D Moralejo D Drey N Chudleigh J Interventions to improve hand hygiene compliance in patient care Cochrane Database of Systematic Reviews 2010 CD005186 10.1002/14651858.CD005186.pub3 20824842

[B21] Gould DJ Chudleigh JH Moralejo D Drey N Interventions to improve hand hygiene compliance in patient care Cochrane Database of Systematic Reviews 2007 10.1002/14651858.CD005186.pub2 17443575

[B22] Fuller C Michie S Savage J McAteer J Besser S Charlett A Hayward A Cookson BD Cooper BS Buckworth G Jeanes A Roberts J Teare L Stone S The Feedback Intervention Trial (FIT)—Improving Hand-Hygiene Compliance in UK Healthcare Workers: A Stepped Wedge Cluster Randomised Controlled Trial PLoS One 2012 7 http://www.plosone.org/article/info%3Adoi%2F10.1371%2Fjournal.pone.0041617. 10.1371/journal.pone.0041617 PMC3479093 23110040

[B23] Chagpar A Banez C Lopez R Cafazzo JA Challenges of Hand Hygiene in Healthcare: The development of a tool kit to create supportive processes and environments Healthcare Quarterly 2010 13 59 66 20959732 10.12927/hcq.2010.21968

[B24] Haas J Larson EL Compliance with Hand Hygiene Am J Nurs 2008 108 40 44 18664758 10.1097/01.NAJ.0000330260.76229.71

[B25] Boscart VM Fernie GR Lee JH Jaglal SB Using psychological theory to inform methods to optimize the implementation of a hand hygiene intervention Implement Sci 2012 7 77 10.1186/1748-5908-7-77 22929925 PMC3503739

[B26] Pyne E Physician Attitudes toward Hand Hygiene in the Acute Care Setting 2012 http://www.dhs.wisconsin.gov/communicable/HAI/Reports/HHProjectReport.pdf.

[B27] Wharton K Karlowicz G Barriers to full compliance with hand-washing guidelines in a neonatal intensive care unite 1487 Pediatr Res 1998 43 254

[B28] Askarian M Mirzaei K Mundy LM McLaws ML Assessment of knowledge, attitudes, and practices regarding isolation precautions among Iranian healthcare workers Infection Control & Hospital Epidemiology 2005 26 105 108 10.1086/502495 15693417

[B29] Stein AD Makarawo TP Ahmad MF A survey of doctors’ and nurses’ knowledge, attitudes and compliance with infection control guidelines in Birmingham teaching hospitals J Hosp Infect 2003 54 68 73 10.1016/S0195-6701(03)00074-4 12767850

[B30] Evanoff B Kim L Mutha S Jeffe D Haase C Andereck D Fraser V Compliance with universal precautions among emergency department personnel caring for trauma patients Ann Emerg Med 1999 33 160 165 10.1016/S0196-0644(99)70389-6 9922411

[B31] Pittet D Simon A Hugonnet S Pessoa-Silva CL Sauvan V Perneger TV Hand hygiene among physicians: performance, beliefs, and perceptions Ann Intern Med 2004 141 1 8 15238364 10.7326/0003-4819-141-1-200407060-00008

[B32] Berhe M Edmond MB Bearman GM Practices and an assessment of health care workers’ perceptions of compliance with infection control knowledge of nosocomial infections Am J Infect Control 2005 33 55 57 10.1016/j.ajic.2004.07.011 15685137

[B33] Tibballs J Teaching hospital medical staff to handwash The Medical Journal of Australia 2012 164 395 398 8609848 10.5694/j.1326-5377.1996.tb124899.x

[B34] Kelen GD Green GB Hexter DA Fortenberry DC Taylor E Fleetwood DH Sivertson KT Substantial improvement in compliance with universal precautions in an emergency department following institution of policy Arch Intern Med 1991 151 2051 2056 10.1001/archinte.1991.00400100121020 1929694

[B35] Moore S Goodwin H Grossberg R Toltzis P Compliance with universal precautions among pediatric residents Archives of Pediatrics & Adolescent Medicine 1998 152 554 557 9641708 10.1001/archpedi.152.6.554

[B36] Jang JH Wu S Kirzner D Moore C Tong A McCreight L Stewart R Green K McGeer A Physicians and hand hygiene practice: a focus group study J Hosp Infect 2010 76 87 89 10.1016/j.jhin.2010.04.021 20638748

[B37] Lankford MG Zembower TR Trick WE Hacek DM Noskin GA Peterson LR Influence of role models and hospital design on hand hygiene of healthcare workers Emergency Infectious Diseases 2003 9 217 223 10.3201/eid0902.020249 PMC2901948 12603993

[B38] Cantrell D Shamriz O Cohen MJ Stern Z Block C Brezis M Hand hygiene compliance by physicians: marked heterogeneity due to local culture? Am J Infect Control 2009 37 301 305 10.1016/j.ajic.2008.05.001 18834749

[B39] Craig P Dieppe PA Macintyre S Michie S Nazareth I Petticrew M Developing and evaluating complex interventions: the new Medical Research Council guidance Br Med J 2008 337 979 983 10.1136/bmj.a979 PMC2769032 18824488

[B40] Campbell M Fitzpatrick R Haines A Kinmonth AL Sandercock P Spiegelhalter D Tyrer P Framework for design and evaluation of complex interventions to improve health BMJ 2000 321 694 696 10.1136/bmj.321.7262.694 10987780 PMC1118564

[B41] Davies P Walker AE Grimshaw JM A systematic review of the use of theory in the design of guideline dissemination and implementation strategies and interpretation of the results of rigorous evaluations Implement Sci 2010 5 http://www.ncbi.nlm.nih.gov/pmc/articles/PMC2832624/pdf/1748-5908-5-14.pdf. 10.1186/1748-5908-5-14 PMC2832624 20181130

[B42] Sandelowski M Sample size in qualitative research 1995 18 179 183 10.1002/nur.4770180211 7899572

[B43] Francis J Johnston M Robertson C Glidewell L Entwistle V Eccles M Grimshaw J What is an adequate sample size? Operationalising data saturation for theory-based interview studies Psychol Heal 2010 25 1229 1245 10.1080/08870440903194015 20204937

[B44] Cane J O’Connor D Michie S Validation of the theoretical domains framework for use in behaviour change and implementation research Implement Sci 2012 7 37 10.1186/1748-5908-7-37 22530986 PMC3483008

[B45] Michie S Johnston M Abraham C Lawton R Parker D Walker A Making psychological theory useful for implementing evidence based practice: a consensus approach Quality and Safety in Health Care 2005 14 26 33 10.1136/qshc.2004.011155 15692000 PMC1743963

[B46] French S Green S O’Connor D McKenzie J Francis J Michie S Buchbinder R Schattner P Spike N Grimshaw J Developing theory-informed behaviour change interventions to implement evidence into practice: a systematic approach using the Theoretical Domains Framework Implement Sci 2012 7 38 10.1186/1748-5908-7-38 22531013 PMC3443064

[B47] Morse JM Field PA Qualitative research methods for health professionals 1995 Thousand Oaks, CA: Sage

[B48] Marshall C Rossman G Designing Qualitative Research 1989 Newbury Park: Sage

[B49] Crabtree B Miller W Doing Qualitative Research 1992 Newbury Park: Sage

[B50] QSR International NVivo10 2012 http://www.qsrinternational.com/products_nvivo.aspx.

[B51] Patey A Islam R Francis JJ Bryson GL Grimshaw JM Anesthesiologists’ and surgeons’ perceptions about routine preoperative testing in low-risk patients: Application of the Theoretical Domains Framework to identify factors that influence physicians’ decisions to order pre-operative tests Implemenation Science 2012 7 http://www.ncbi.nlm.nih.gov/pmc/articles/PMC3522997/pdf/1748-5908-7-52.pdf. 10.1186/1748-5908-7-52 PMC3522997 22682612

[B52] Islam R Tinmouth AT Francis JJ Brehaut JC Born J Stockton C Stanworth SJ Eccles MP Cuthbertson BH Hyde C Grimshaw JM A cross-country comparison of intensive care physicians’ beliefs about their transfusion behaviour: A wualitative study using the theoretical domains framework Implement Sci 2012 7 http://www.ncbi.nlm.nih.gov/pmc/articles/PMC3527303/pdf/1748-5908-7-93.pdf. 10.1186/1748-5908-7-93 PMC3527303 22999460

[B53] Thompson C McCaughan D Cullum N Sheldon TA Raynor P Increasing the visibility of coding decision in team-based qualitative research Int J Nurs Stud 2004 41 15 20 10.1016/j.ijnurstu.2003.03.001 14670390

[B54] Miles MB Huberman AM Qualitative Data Analysis: An Expanded Sourcebook 1994 Thousand Oaks, CA: Sage

[B55] Francis JJ Stockton C Eccles MP Cuthbertson BH Grimshaw JM Hyde C Evidence based selection of theories for designing behaviour change interventions: Using methods based on theoretical construct domains to understans clinicians’ blood transfusion behaviour Br J Health Psychol 2009 14 625 646 10.1348/135910708X397025 19159506

[B56] Zhang Y Wildemuth BM Qualitative analysis of content Applications of Social Research Methods to Questions in Information and Library Science 2009 Westport, CT: Libraries Unlimited 308 319

[B57] Patton MQ Qualitative evaluation and research methods 1990 Thousand Oaks, California: Sage Publications, Inc 2

[B58] van Bokhoven MA Kok G Van der Weijden T Designing a quality improvement intervention: a systematic approach Quality and Safety in Health Care 2003 12 215 220 10.1136/qhc.12.3.215 12792013 PMC1743716

[B59] Michie S Johnston M Francis J Hardeman W Eccles M From theory to intervention: mapping theoretically derived behavioural determinants to behaviour change techniques Appl Psychol 2008 57 660 680 10.1111/j.1464-0597.2008.00341.x

[B60] Bloor M Franskland J Thomas M Robson K Focus groups in social research 2001 London: Sage

