# Supplementary material for: Improving physician hand hygiene compliance using behavioural theories: a study protocol
Source: Implement Sci. 2013 Feb 4;8:16. doi: 10.1186/1748-5908-8-16 (PMC3571966; doi:10.1186/1748-5908-8-16)
Supplement: Additional file 1 — Interview Guide. [file 1748-5908-8-16-S1.doc]

**Additional File 1 -** Interview Guide

***Explanation***

Thank you for agreeing to speak with me today about hand hygiene practice. There may appear to be overlap between questions but each question is worded to obtain specific information and therefore you may find that answers are repeated. It is important to note that there are no right or wrong answers to the questions and that no one will know what your specific answers were.

The interview should take approximately 15 to 20 minutes and will be audio-recorded to ensure that all key points are accurately documented. Any identifying information (for example the names of other individuals) that you use in the course of our discussion will be removed from the interview transcripts. If you wish to end the interview before I have asked all of the questions or if you wish to withdraw from the study you are free to do so.

Background

- - Male or Female (to keep track of, won’t be asked)
  - Confirm if right campus documented (for staff physicians)
  - Age range- <30, 31-40, 41-50, 51-60, 61+
  - How long have you been a physician?
  - What year of residency? (if applies)
  - How long have you been at TOH?
  - Have you done any of your training at TOH in the past 10 years (medical school, residency, fellowship)?

1. Knowledge
   - Are you aware of any guidelines about hand hygiene?
     1. If yes, what are they?
     2. Are you familiar with the 4 moments of hand hygiene?
   - Are you aware of any evidence that links hand hygiene to healthcare associated infections?
     1. If yes, what are your thoughts about this evidence? (prompt: do you agree? etc.)
   - Do you feel as though you have a sufficiently strong background in infection control training?
     1. If yes, what is the training?
     2. If no, what would you like to know more about?

- When do you think that you need to practice hand hygiene?

1. Skills
   - Were you ever trained in the proper technique for hand hygiene?
   - Do you think proper hand hygiene is a skill?
2. Social/Professional Role and Identity
   - Is hand hygiene a standard part of your patient consultations?

- Is hand hygiene something specific to [residents] [staff physicians] [surgeons] [general medicine]?
- What is your impression of the compliance of others in your profession with hand hygiene guidelines? Do you feel as though your hand hygiene practice is in line with your peers?

1. Beliefs about Capabilities
   - How easy or difficult is it for you to practice hand hygiene? What made it easy what made it difficult?
   - Do you believe that what you consider to be good hand hygiene fits with the current guidelines?
   - Are you confident that you are following the guidelines when practicing hand hygiene?
2. Beliefs about consequences
   - What are the benefits when good hand hygiene is practiced? (prompts: patients, yourself)

- What are the negative aspects when good hand hygiene is practiced? (prompts: patients, yourself)
  - In what situations do you think hand hygiene is necessary/unnecessary?

1. Optimism
   - What do you think will happen if you are not able to practice hand hygiene (prompt: the 4 moments)?
   - In your opinion, how likely is it that improper hand hygiene will lead to a healthcare associated infection?
2. Intentions
   - Do you intend to practice hand hygiene (prompt: the 4 moments)?
     1. If no, why?
     2. If yes, do you anticipate any problems?

- What will make the process of hand hygiene easier?

1. Memory, Attention and Decision Processes
   - Is hand hygiene automatic or do you need to remember or be reminded to do it?
   - Is practicing or not practicing hand hygiene ever a conscious decision?
   - Do you have any triggers for remembering to practice hand hygiene? What would make it easier to remember hand hygiene?
   - Are there certain situations where you find yourself forgetting to practice hand hygiene more often than others?
   - In what situations might you find it difficult to follow the hand hygiene guidelines?
2. Environmental context and resources
   - Have you found hand hygiene audits to be successful at promoting compliance? If yes, what aspects of this intervention encourage hand hygiene? If no, what could be done to improve this intervention?
   - What resources would make it easier for you to practice hand hygiene?
   - What aspects of your work environment influence whether you practice hand hygiene? (PROMPT: any trigger or prompts in clinic?)
   - Are there any competing tasks or time constraints that might influence whether or not you practice hand hygiene? What would help you overcome these problems/difficulties?
3. Social influences
   - Do other team members influence your decision to practice hand hygiene?
     1. If yes, how?
     2. If no, prompt (co-workers / team lead / department / overall workplace)
   - Do the expectations of your patients and their families influence you to practice hand hygiene?
     1. If yes, how?
4. Emotion
   - Do you have any strong feelings about current hand hygiene guidelines (prompt: 4 moments)?
   - Do your emotions or mood ever influence whether you practice hand hygiene?
   - Does not practicing hand hygiene evoke worry or concern in you?
5. Goals

- Do you want to practice hand hygiene (prompt: the 4 moments)?
- In what situations do you want to practice hand hygiene?
- Considering your other priorities, on a scale of 1 to 10 with 10 being very important, how important do you think it is for you to practice hand hygiene (prompt: the 4 moments)?
  - 1. (If not 10), what is a higher priority?

1. Behavioural regulation
   - What could you personally do to increase your hand hygiene practice? (prompt: within your everyday practice)
   - What do you think is needed to ensure that you consistently practice hand hygiene?
   - Within your healthcare team Are there procedures or ways of working that encourage hand hygiene?
2. Reinforcement

- In the past, are there any personal or external incentives that you have experienced to be effective to improve hand hygiene? (prompt: yourself, or others)
- Have your views toward hand hygiene evolved from your time in medical school up until now?

1. If so, how?
